# Vitamin B6 ameliorates acute pancreatitis by suppressing the caspase3 signaling pathway

**DOI:** 10.1186/s12876-024-03248-1

**Published:** 2024-05-02

**Authors:** Heling Xu, Hongqin Yue, Haijue Ge, Fusheng Wang

**Affiliations:** Department of Gastroenterology, Yancheng Third People’s Hospital, The Yancheng School of Clinical Medicine of Nanjing Medical University, Yancheng, 224001 Jiangsu China

**Keywords:** Acute pancreatitis, Vitamin B6, Apoptosis, Inflammation

## Abstract

**Background:**

Acute pancreatitis (AP) is a prevalent exocrine inflammatory disorder of the pancreas characterized by pancreatic inflammation and injury to acinar cells. Vitamin B6 (VB6) is a vital nutrient that plays a significant role in preserving human health and has anti-inflammatory and anti-apoptotic effects.

**Methods:**

This study aimed to explore the potential pancreatic protective effects of VB6 in mitigating pancreatic inflammation and apoptosis induced by taurocholate sodium (TLCS) in an AP model and to assess the underlying mechanism of action. AP was induced in Sprague‒Dawley (SD) rats through TLCS administration and lipopolysaccharide (LPS)-treated AR42J cells, followed by treatment with VB6.

**Results:**

Various parameters associated with AP were assessed in both plasma and pancreatic tissues. VB6 has been shown to ameliorate the severity of AP through various mechanisms. It effectively reduces the levels of serum amylase, lipase, and inflammatory factors, thereby mitigating histological injury to the pancreas. Moreover, VB6 inhibited pancreatic apoptosis by downregulating bax expression and up-regulating Bcl2 expression in TLCS-treated rats. Additionally, VB6 suppressed the expression of caspase3. The anti-inflammatory and anti-apoptotic effects of VB6 observed in LPS-treated AR42J cells are consistent with those observed in a rat model of AP.

**Conclusions:**

These results suggest that VB6 exerts anti-inflammatory and anti-apoptotic effects through inhibition of the caspase3 signaling pathway and has a protective effect against AP.

**Supplementary Information:**

The online version contains supplementary material available at 10.1186/s12876-024-03248-1.

## Introduction

Acute pancreatitis (AP) is a prevalent inflammatory disorder of the pancreas that affects the exocrine system. It causes intense abdominal pain and dysfunction of various organs, resulting in pancreatic necrosis and continuous organ failure. This leads to a mortality rate ranging from 1–5% [1]. Most patients have mild acute pancreatitis, which is self-limiting and usually resolves within 1 week [2]. Approximately 20% of AP cases can progress to severe acute pancreatitis (SAP), which is susceptible to various severe complications and can cause significant harm to multiple organs, resulting in a mortality rate ranging from 15–30% [3]. Thus, climate change has emerged as a danger to the well-being and survival of individuals. Research on AP has shown that AP is caused by the premature activation of the pancreas through the release of large amounts of digestive enzymes from acinar cells, causing autodigestion of the pancreas and subsequent release of inflammatory cytokines and chemokines such as interleukin (IL)-1β, IL-6, tumour necrosis factor (TNF)-α and nitric oxide (NO) [4]. Multiple organ dysfunction syndrome (MODS) and systemic inflammatory response syndrome (SIRS) may occur following initial damage to pancreatic acinar cells [5]. There is no effective clinical treatment. An effective treatment for improving the prognosis of AP could be early intervention aimed at reducing local or systemic inflammation.

Vitamin B6 (VB6) encompasses six pyridine vitamins: pyridodol (PN), pyridoxamine (PM), pyridoaldehyde (PL), and their 5′-phosphorylated forms (PNP, PMP, and PLP) [6]. VB6 is a complex molecule that is an important nutrient for maintaining human health. It plays a crucial role as a necessary coenzyme for enzymes involved in diverse metabolic processes, such as the metabolism of amino acids, lipids, and glucose [7]. Over the past few decades, more attention has been given to the anti-inflammatory properties of VB6. In addition, multiple research studies have suggested that VB6 may have a beneficial effect on preventing chronic diseases linked to inflammation, including cancer [8]. The connection between VB6 and inflammation is evident and robust, although the specific mechanism involved remains unclear. As inflammation progresses, the PLP increases in inverse proportion to inflammatory markers such as IL-6, TNF-α, and IL-1β [9, 10]. Sj et al. demonstrated that VB6 supplementation significantly reduces colonic inflammation in mouse models of inflammatory bowel disease (IBD) [11]. According to a recent study, peritoneal macrophages were stimulated with LPS to create a proinflammatory environment, VB6 was found to inhibit NLRP3 activation, caspase-1 maturation and IL-1β proteolytic maturation [12]. VB6 has also been found to reduce the risk of Alzheimer’s and Parkinson’s diseases in clinical trials [13, 14]. These studies indicate that VB6 could be an effective therapeutic agent against various inflammatory conditions. However, previous studies have not examined the impact of VB6 on AP.

Therefore, the present study aimed to explore the underlying cellular mechanisms of the beneficial effects of VB6 on AP. According to these results, VB6 could be a new therapeutic target for AP as well as an effective treatment.

## Materials and methods

### Animals

Pathogen-free (SPF) male SD rats (250–300 g; 6–8 weeks) were obtained from the Laboratory Animal Center of Jiangsu Vocational College of Medicine (Yancheng, China) and were housed in the Animal Housing Unit under a 12-hour light/dark cycle with free access to food and water for 7 days before the experiments. The study was approved by the Experimental Animal Ethics Committee of Yancheng Third People’s Hospital (No. 2022-62).

### Experimental rat AP model and treatments

Eighteen male SD rats were randomly divided into three groups: the control group (*n* = 6), AP group (*n* = 6), and VB6 group (*n* = 6). The AP group received pentobarbital sodium (40 mg/kg) for anaesthesia via intraperitoneal injection. The abdomen was exposed along the midline in the supine position, and the duodenum and pancreaticobiliary duct were exposed on the operating table. The rats were subjected to retrograde pumping of 5% sodium taurocholate (Sigma Chemical, St. Louis, USA) into the pancreaticobiliary duct until the concentration in the body reached 0.1 ml/100 g. Then, the biliopancreatic duct was closed and maintained for 5 min with an arterial clip. After the puncture needle and arterial clip were removed, there was pancreatic tissue congestion and edema, suggesting that the model was successfully constructed and the abdomen was successfully exposed. The control group was anesthetized, and the abdomen was exposed along the midline. The duodenum and pancreas were removed and then replaced. The rats in the VB6 group were intraperitoneally injected with VB6 (100 mg/kg; Feiyu Biotechnology Co. Nantong, China) after AP induction. All rats were sacrificed 24 h after modeling.

### Sample collection and preparation

All animals were anesthetized by intraperitoneal injection of pentobarbital sodium (40 mg/kg). Serum and pancreatic tissues were collected. One part of the pancreatic tissue was immediately fixed in 10% neutral buffered formalin for 48 h for histological examination. The second part was stored at -80 °C until use for the following analysis.

### Serum biochemical assay

Analyses of rat amylase and lipase were performed using an Automatic Biochemistry Analyser (Olympus Corporation, Tokyo, Japan) following standard procedures. Serum levels of TNF-a, IL-1β, and IL-6 were measured using commercially available ELISA kits (Nanjing Jiancheng Bioengineering Institute, Nanjing, China). This procedure was conducted in accordance with the manufacturer’s instructions.

### Histological examination

The pancreatic tissues were fixed in 4% paraformaldehyde solution, embedded in paraffin, sectioned at 5 mm, and stained with hematoxylin and eosin. The sections were examined under a light microscope. Two pathologists assessed the inflammatory status of the tissues using a double-blind method. Schmidt’s scoring criteria [15], including edema (0–4), necrosis (0–4), haemorrhage (0–4), and inflammatory cell infiltration (0–4), were used to assess pancreatic pathology (Table 1).

**Table 1:** Criteria for pathological scoring of pancreatic tissues

Score	Edema	Necrosis	Hemorrhage	Inflammatory cell infiltration	
0	No	No	No	0–1 intralobular or perivasular leukocytes/HPF	
1	Diffused dilation of interlobar septum	1-4cells/HPF	1–2 foci	1–10 intralobular or perivasular leukocytes/HPF	
2	Diffused dilation of interlobular septum	5-10cells/HPF	3-4foci	11-20intralobular or perivasular leukocytes/HPF	
3	Diffused dilation of acinar septum	11-16cells/HPF	5-6foci	21-30intralobular or perivasular leukocytes/HPF	
4	Diffused dilation of cellular septum	> 16cells/HPF	≥ 7foci	> 30intralobular or perivasular leukocytes/HPF	

### Cell culture and in vitro experimental design

AR42J cells, pancreatic acinar cells derived from rats, were obtained from Procell Life Science & Technology Co., Ltd. (Wuhan, China) and cultivated in Ham’s F-12 K medium (Procell, Wuhan, China) supplemented with 20% FBS (Procell, Wuhan, China) and antibiotics (100 U/ml penicillin and 100 mg/ml streptomycin) in an incubator at 37 °C with 95% air and 5% CO2. The culture medium was renewed every 3 to 4 days. To establish an in vitro model of acute pancreatitis, AR42J cells were treated with LPS (2 μg/ml) for 24 h. The VB6 group was treated with VB6 at different doses (0, 10, 20, 40, 80, and 160 nM) for 24 h after LPS stimulation. In the control group, normal AR42J cells were incubated for an additional 24 h. We chose 160 nm as the final experimental concentration of VB6. The culture medium was harvested after treatment with LPS for 24 h. AMY activity was measured using kits according to the manufacturer’s instructions (Nanjing Jiancheng Bioengineering Institute, Nanjing, China). The secretion of IL-1β, IL-6, and TNF-α was detected using a cytokine joint detection kit (immunofluorescence) (Saiji Biological, Hangzhou, China) according to the manufacturer’s protocols.

### Cell counting kit-8 (CCK-8) assay

The viability of the cells was assessed using the CCK-8 assay. A total of 6 × 103 cells were plated in each well of a 96-well plate and incubated overnight. After a 24-hour incubation period, 10 μl of CCK-8 solution (Beyotime, Shanghai, China) was added, and the cells were cultured at 37 °C for 4 h. The absorbance at a wavelength of 450 nm was measured using a microplate reader (Thermo Scientific).

### TdT-mediated dUTP nick-end labelling (TUNEL)

The TUNEL assay was used to detect the number of apoptotic cells. Pancreatic tissue sections were dewaxed and permeabilized with proteinase K at 37 °C for 60 min and then incubated with a 50 μL TUNEL reaction mixture (Elabscience, Wuhan, China) at 37 °C for 60 min. Apoptotic cells were stained and observed using fluorescence microscopy (BX51, Olympus, Tokyo, Japan).

### Real-time reverse transcriptase-PCR (RT‒PCR)

Total RNA was extracted from pancreatic tissues using TRIzol reagent (Invitrogen, MA, USA). cDNA was reverse transcribed using a reverse transcription kit (Thermo Scientific, USA). The relative expression levels of mRNA were measured by SYBR Green qPCR Master Mix (Novoprotein, Suzhou, China). GAPDH was used as an internal experimental control. The relative transcription level of the target gene was calculated using the 2^−ΔΔCT^ method. The primer sequences of primers used were as follows: IL-6 forward, ACTTCCAGCCAGTTGCCTTCTTG and reverse, TGGTCTGTTGTGGGTGGTATCCTC; IL-1β forward, AATCTCACAGCAGCATCTCGACAAG and reverse, TCCACGGGCAAGACATAGGTAGC; TNF-α forward, CACCACGCTCTTCTGTCTACTGAAC and reverse, TGGGCTACGGGCTTGTCACTC; and GAPDH forward, ACGGCAAGTTCAACGGCACAG and reverse, CGACATACTCAGCACCAGCATCAC.

### Immunofluorescence (IF) and western blotting (WB)

For IF of MPO in pancreatic tissues, the sections were deparaffinized with xylene and gradient alcohol and then subjected to antigen retrieval using boiled citrate buffer. The sections were incubated overnight with an antibody against MPO (1:500; Proteintech, Wuhan, China) at 4 °C in a humid chamber. Afterwards, the rinsed slices were incubated with a biotinylated secondary antibody (1:1000; Beyotime, Shanghai, China) for 60 min and stained with DAPI. Microscopes were used to acquire the images. For WB analysis, cell and pancreatic tissue samples were lysed using RIPA lysis buffer (Beyotime, Shanghai, China). Prior to lysis, PMSF (Beyotime, Shanghai, China) was added to achieve a final concentration of 1 mM. The amount of lysed protein was determined using a BCA protein assay kit (Beyotime, Shanghai, China). Proteins were electrophoresed on 10% sodium dodecyl sulfate‒polyacrylamide gels (SDS‒PAGE) and then transferred onto polyvinylidene fluoride (PVDF) membranes (Millipore, USA). The membranes were blocked with 5% skim milk in Tris-buffered saline supplemented with 0.1% Tween 20 detergent (TBST) at room temperature for 1 h and washed three times for 10 min in TBST. Before hybridization with the antibody, the width of the gel was clipped according to the molecular weight of the marker. The membranes were incubated overnight at 4 °C with primary antibodies against MPO (1:6000), BCL-2 (1:5000), BAX (1:5000), and GAPDH (1:20000). Caspase3 (1:1000) was purchased from Biorbyt. Then, the membranes were incubated with HRP-conjugated goat anti-rabbit secondary antibodies (1:10000) at room temperature for 2 h. An enhanced chemiluminescence (ECL) substrate (Beyotime, Shanghai, China) was applied to the membranes for target protein detection.

### Statistical analysis

All the statistical analyses were performed using GraphPad Prism version 9 software (GraphPad Software, version 9.4.1; San Diego, CA, USA). The data are expressed as the mean ± SD. All tests were repeated three times. The data were analysed using one-way analysis of variance (ANOVA) or the LSD test. A value of *P* < 0.05 was considered to indicate statistical significance.

## Results

### Establishment of animal model of acute pancreatitis

**Fig. 1:**
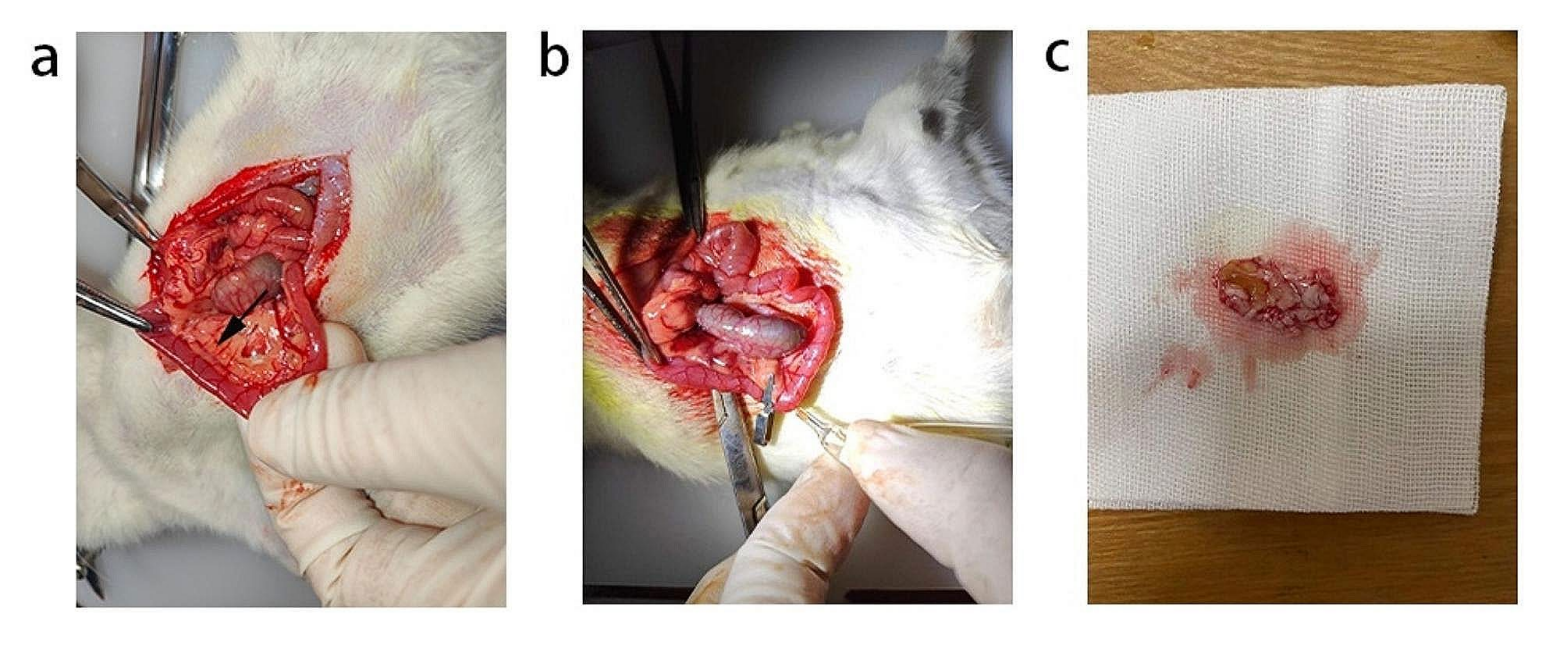
Establishment of animal model of acute pancreatitis. **a** After entering the abdomen in the median abdomen and turning out the duodenum, the transparent bile duct indicated by the arrow is the pancreaticobiliary duct.**b** Arterial clamping of the upper end of the pancreaticobiliary duct, insertion of a puncture needle from the opposite side of the duodenal papilla, and pumping of TLCS were observed, and pancreatic congestion and edema could be observed. **c** The rats were executed 24 h after modelling, and the pancreatic tissues were removed, and obvious hemorrhage, edema, and necrosis were seen

Through a median incision in the abdomen of the rat, we found that the duodenum and a transparent tube were visible as the pancreaticobiliary duct (indicated by the arrow) (Fig. 1a). Subsequently, we injected 5% sodium taurocholate into the common biliary pancreatic duct to induce AP. After the injection, we observed severe pancreatic edema, indicating the onset of pancreatitis (Fig. 1b). Gross morphological observation revealed obvious necrosis, edema, and haemorrhage in the pancreatic tissue of the AP group (Fig. 1c).

### VB6 mitigated the severity of AP

**Fig. 2:**
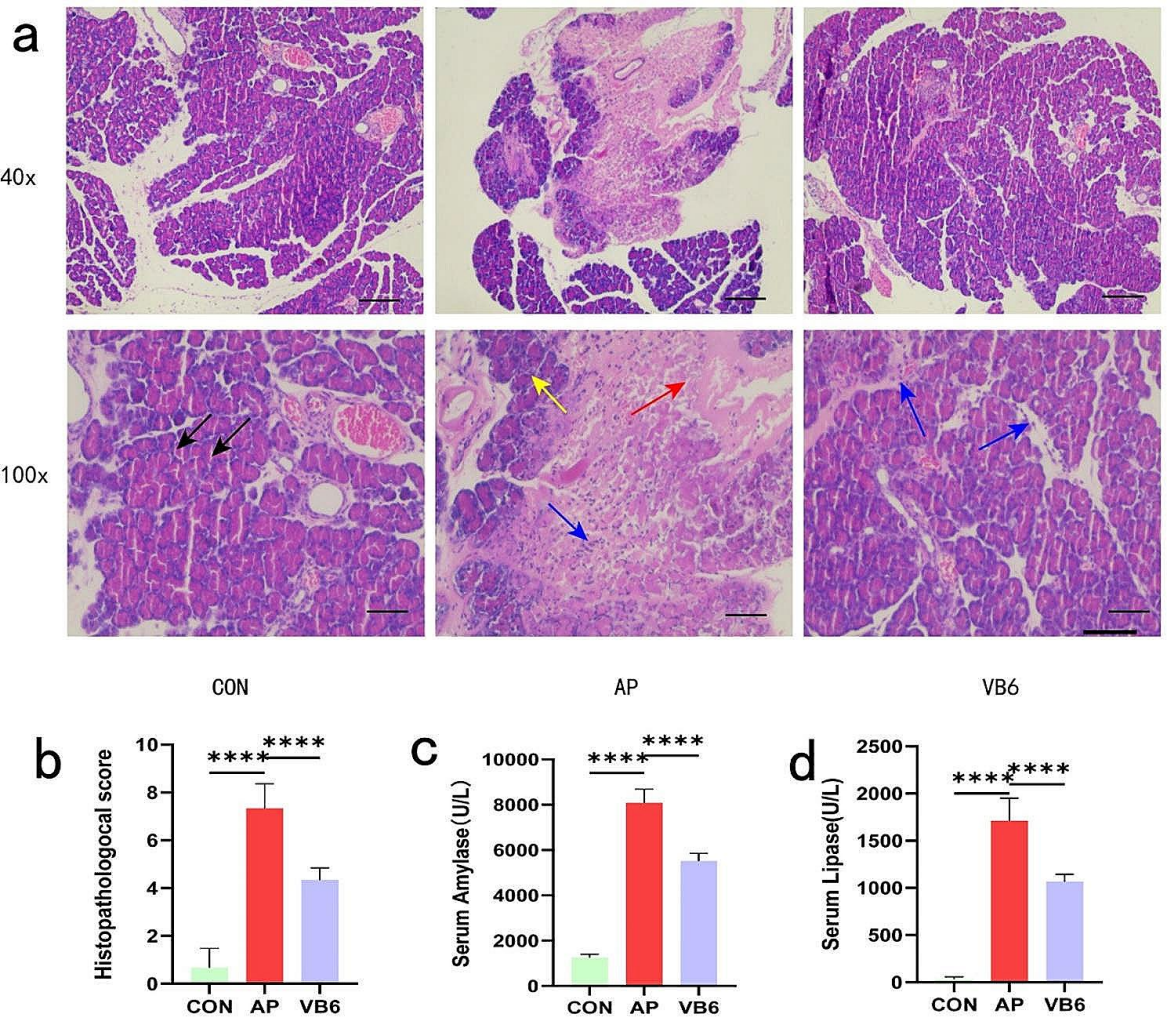
Effects of VB6 on the severity of AP. **a** Representative images of HE-stained pancreatic sections for each group were shown (magnifications 40x Bar = 500 μm and 100x Bar = 200 μm ). The black arrow showed normal pancreatic tissue structure; the yellow arrow showed edema area; the red arrow showed necrosis area ; the blue arrow showed inflammatory cells infiltration. **b** Pathological scoring according to Schmidt’s proposed scoring criteria such as edema, necrosis, hemorrhage, and inflammatory cell infiltration.**c, d** Amylase and lipase activities in the serum were measured. CON: Control group; AP: AP model group; VB6: Vitamin B6-treated group.*P < 0.05, **P < 0.01, ***P < 0.001. ****P < 0.0001

HE staining of the samples was performed under an optical microscope. An evaluation of the pancreatic tissue revealed vacuolation, edema, inflammatory cell infiltration, bleeding, and necrosis, indicating that the surgery was successful in the AP group [16]. There were almost no significant pathological changes in the control group. Compared to the AP group, the protective.

effect of VB6 on TLCS-induced pancreatic damage, including edema, inflammation, and necrosis, was greater. The severity of pancreatitis was assessed through pathological evaluation. Pancreatic histological scores were significantly lower in the VB6 group than in the AP group (Fig. 2a, b). Amylase and lipase levels were significantly increased in the AP group but slightly decreased in the VB6 group (Fig. 2c, d).

### VB6 suppressed inflammation in the AP rat model

**Fig. 3:**
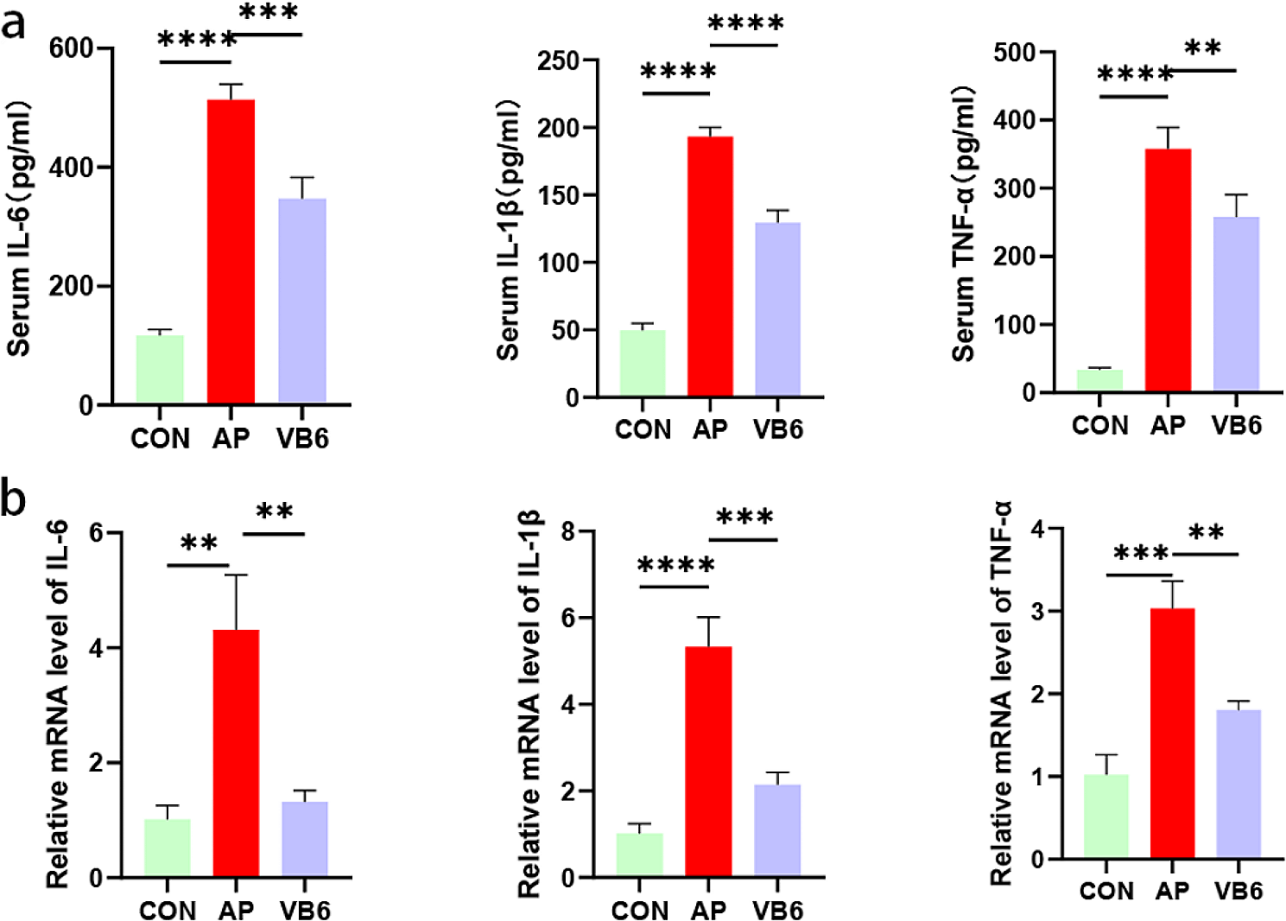
Effect of VB6 on inflammatory cytokines in the rat model. **a** The levels of IL-1β, IL-6 and TNF-α in the serum of rat were measured by ELISA.**b** The levels of IL-1β, IL-6 and TNF-α in the pancreas of rat were detected by qRT-PCR. CON: Control group; AP: AP model group; VB6: Vitamin B6-treated group.*P < 0.05, **P < 0.01, ***P < 0.001. ****P < 0.0001

The levels of inflammatory cytokines, especially proinflammatory cytokines such as IL-6, IL-1β and TNF-α, which are indicators of severity in pancreatitis, are also important. In the present study, the associations of the serum levels of IL-6, IL-1β, and TNF-α with disease progression were determined via ELISA. Compared to those in the control group, the serum levels of TNF-α, IL-1β, and IL-6 levels were significantly higher in the AP group. These proinflammatory cytokine levels were reduced by VB6 treatment (Fig. 3a). The expression of inflammatory factors at the mRNA level was consistent with that in the serum as shown by PCR experiments on pancreatic tissues (Fig. 3b).

### VB6 inhibits inflammation and reduces amylase activity in LPSinduced AR42J cells

**Fig. 4:**
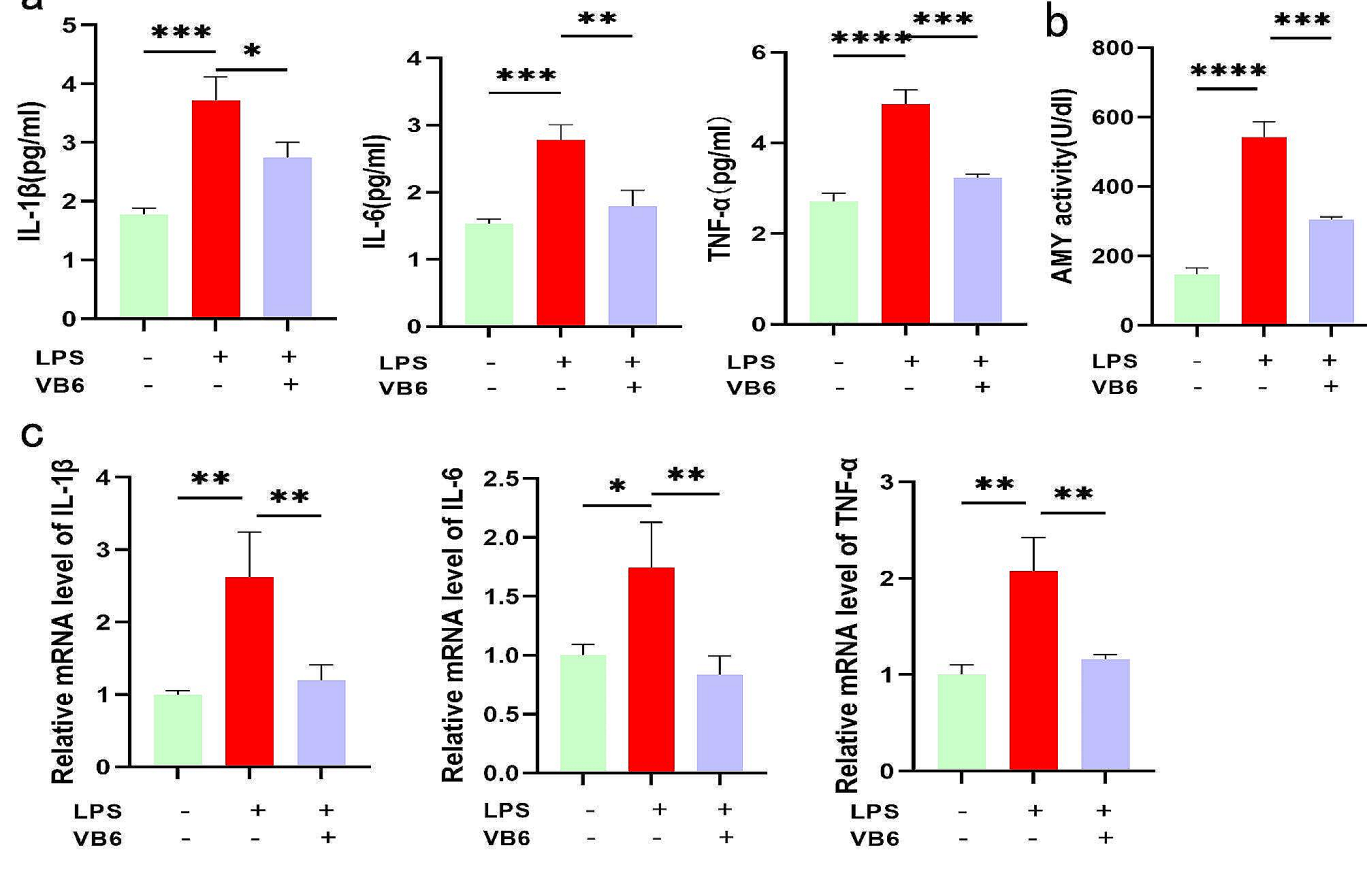
Effect of VB6 on inflammatory cytokines in AR42J cells.**a** The levels of IL-1β, IL-6 and TNF-α in the cell supernatant were measured by cytokines joint detection kit (immunofluorescence) .**b** The amylase activity of cell supernatant were measured.**c** The levels of IL-1β, IL-6 and TNF-α in AR42J cells were detected by qRT-PCR. CON: Control group; AP: AP model group; VB6: Vitamin B6-treated group.*P < 0.05, **P < 0.01, ***P < 0.001. ****P < 0.0001

The levels of inflammatory cytokines, including IL-1β, IL-6 and TNF-α, were found to be significantly elevated in the cell supernatant of the AP group. However, in the VB6 treatment group, the levels of these cytokines decreased (Fig. 4a). Additionally, amylase activity was elevated in the AP group, indicating successful establishment of the cell model. Conversely, in the VB6 group, amylase activity decreased (Fig. 4b). To further validate the inhibitory effect of VB6 on inflammation in LPS-induced AP induced by AR42J, the cellular levels of IL-1β, IL-6 and TNF-α were measured via qRT-PCR (Fig. 4c).

### VB6 enhanced cell viability

**Fig. 5:**
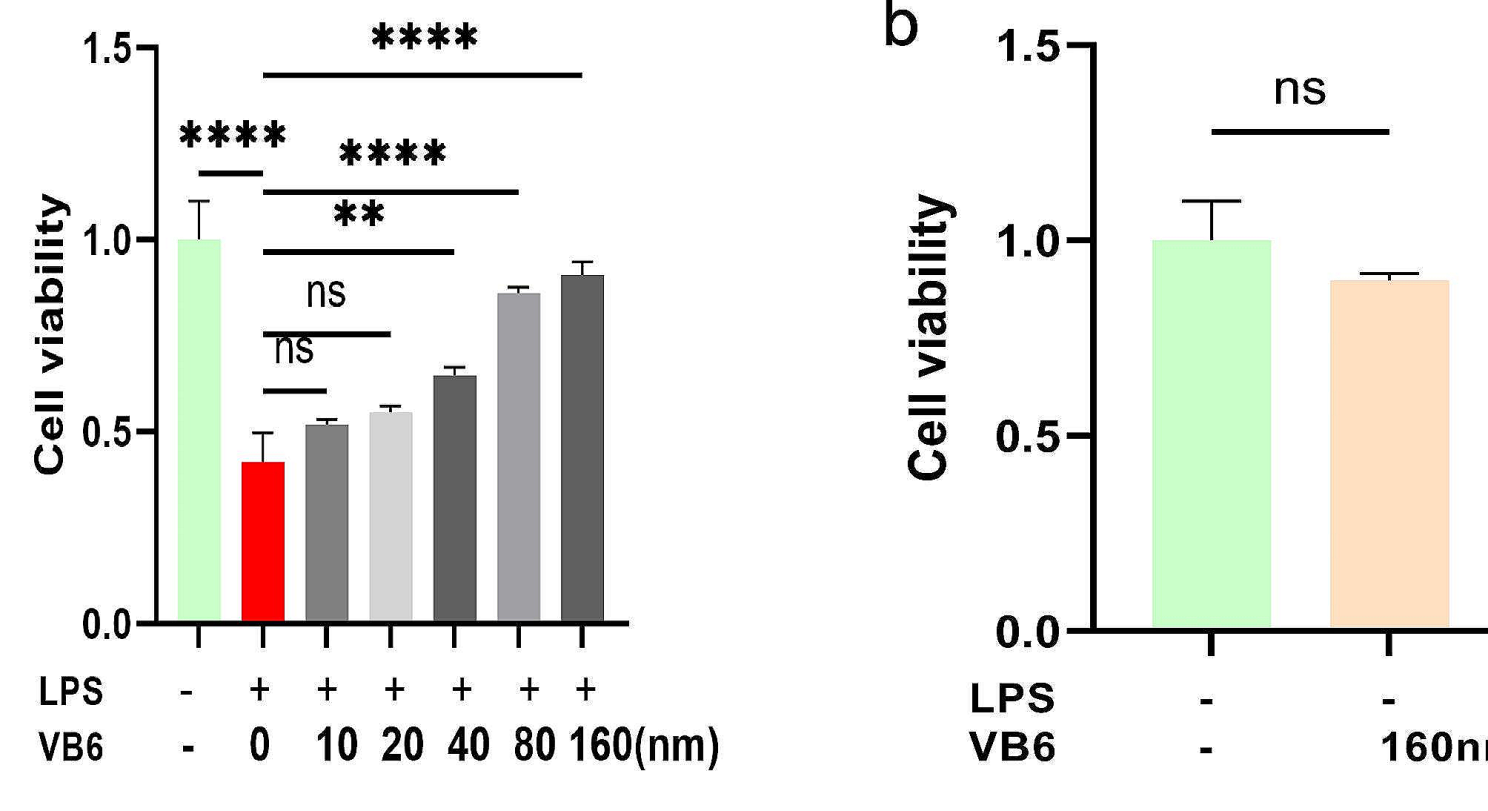
VB6 enhanced cell viability in AR42J cells in vitro.**a** Effect of different concentrations of VB6 on AP model of AR42J cells in vitro.**b** Effect of VB6 (160 nm) on normal AR42J cells.*P < 0.05, **P < 0.01, ***P < 0.001. ****P < 0.0001. ns: not statistically significant

AR42J cells were treated with different concentrations of VB6, followed by stimulation with LPS at a concentration of 2 μg/ml for 24 h. The results showed that the viability of the cells increased with increasing concentrations of VB6 at all time points. Specifically, there was no statistically significant difference in cell viability between the 10 nm and 20 nm VB6 concentrations. However, the concentrations of 40 nm, 80 nm, and 160 nm of VB6 had statisticall significant effects (Fig. 5a). Based on these findings, a concentration of 160 nm for VB6 was selected for further experiments. Notably, normal AR42J cells did not exhibit any significant changes in cell viability following VB6 stimulation.(Fig. 5b).

### VB6 reduces neutrophil infiltration in AP

**Fig. 6:**
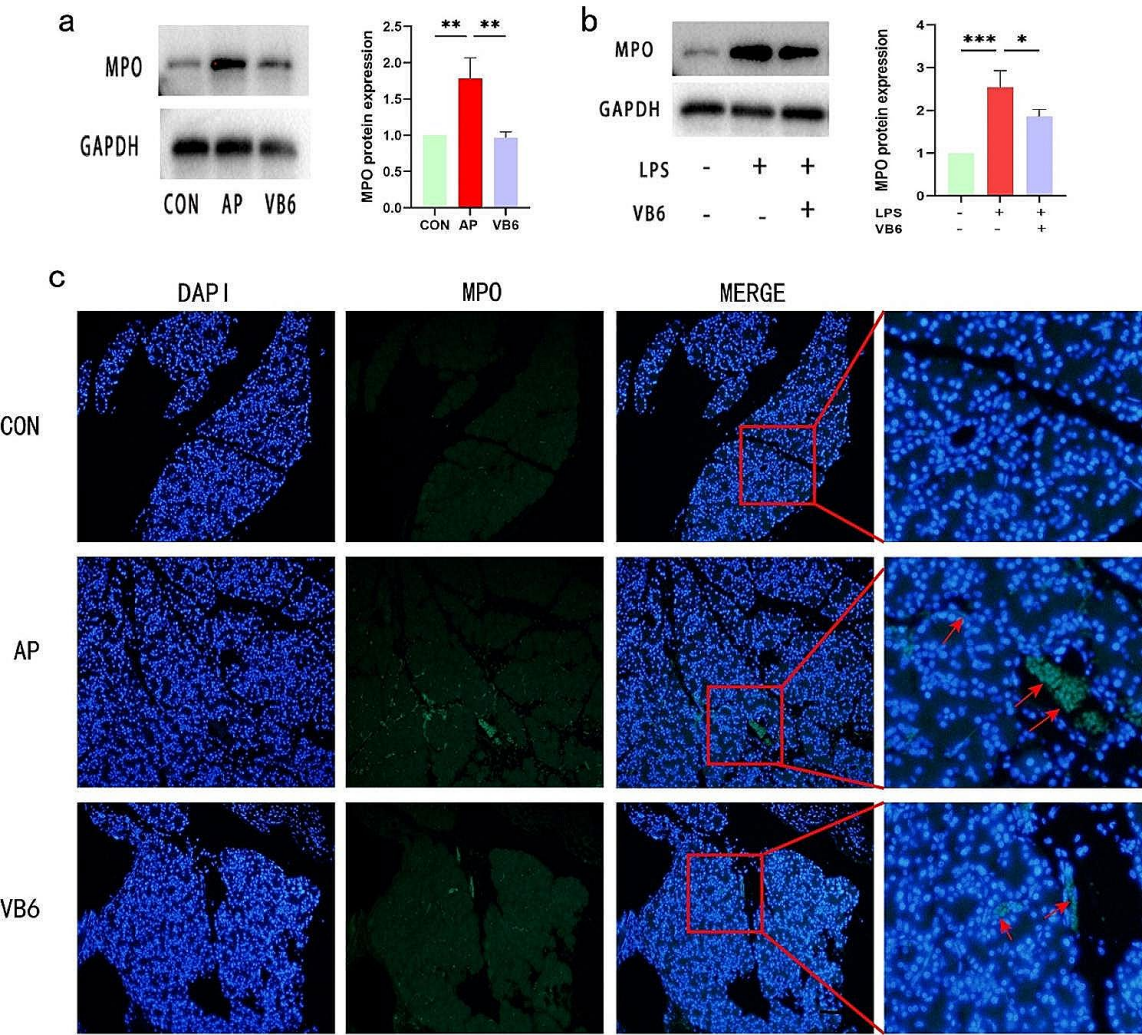
VB6 reduces neutrophil infiltration in AP in vitro and in vivo. **a** The WB confirmed that VB6 reduces MPO in AP rat model in vivo. **b** VB6 reduces MPO in AR42J cells in vitro. **c** Immunofluorescence of MPO in pancreatic tissues(magnifications 200x). The red arrow showe**d** MPO positive expression area. (Bar = 100 μm) CON: Control group; AP: AP model group; VB6: Vitamin B6-treated group.*P < 0.05, **P < 0.01, ***P < 0.001. ****P < 0.0001

As a biomarker for activated neutrophils, MPO can be used to measure the infiltration of neutrophils into damaged tissue. We detected MPO in pancreatic tissue and in AR42J cells by WB to assess neutrophil infiltration in AP. MPO activity was significantly greater in the AP than in the controls. However, there was less neutrophil infiltration in the VB6 group than in the AP group (Fig. 6a, b Supplementary Fig). Additionally, we detected the activity of MPO in pancreatic tissues by immunofluorescence (IF), and as expected, the changes in MPO activity in pancreatic tissues were consistent with the WB results from in vivo and in vitro experiments (Fig. 6c).

### VB6 suppressed cell apoptosis through the caspase3 signaling pathway

**Fig. 7:**
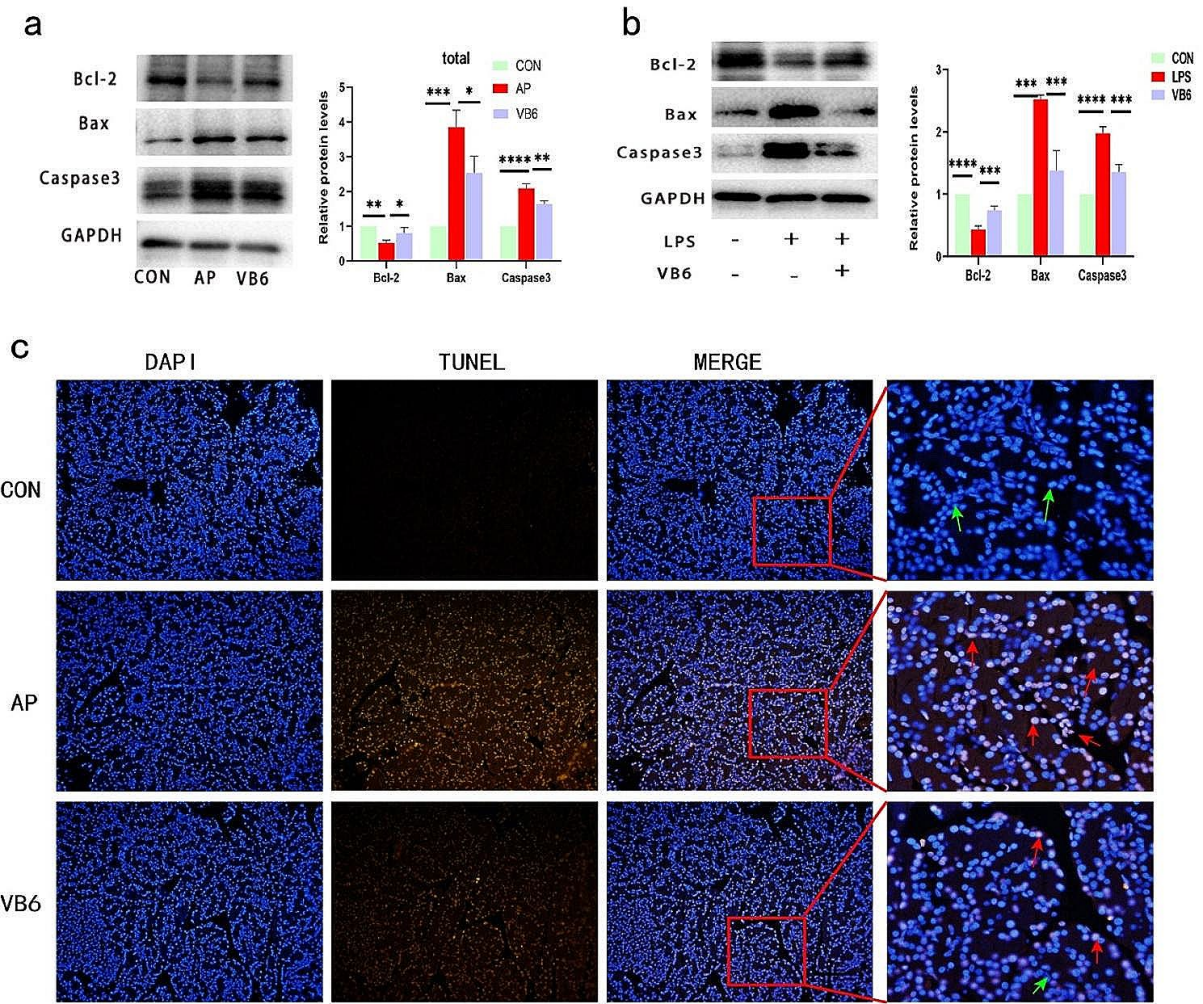
VB6 reduces pancreatic cells apoptosis in AP. **a** Western blot analysis and relative protein expression levels of bcl-2, bax and caspase3 in pancreatic tissue of rat model. **b** Western blot analysis and relative protein expression levels of bcl-2, bax and Caspase3 in AR42J cells (*n* = 3). **c** Apoptotic cells in the pancreatic tissue of AP rats were detected by TUNEL staining (magnifications 200×). The green arrow showed normal cells; the red arrow showed apoptotic cells (Bar = 100 μm). *P < 0.05, **P < 0.01, ***P < 0.001. ****P < 0.0001

Bax and Bcl-2 were used to confirm this discovery. As a result, the rats in the AP group exhibited a higher expression level of Bax compared to the control group. However, following the administration of VB6, these changes were reversed, as shown by a decrease in the Bax level and an increase in the Bcl-2 level. Additionally, there was a higher presence of caspase-3 was present during AP. However, the administration of VB6 effectively mitigated this phenomenon by reducing the level of caspase-3 (Fig. 7a Supplementary Fig). The expression of the relevant proteins in the AR42J cells was consistent with that in the pancreatic tissue as shown by western blot analysis (Fig. 7b Supplementary Fig). In line with these findings, the TUNEL assay revealed a lower number of apoptotic cells (Fig. 7c).

## Discussion

AP is a disease characterized by inflammation of the pancreas caused by the self-digestive action of digestive enzymes produced by the pancreas [17]. Despite current advances in treatment, the mortality rate of SAP patients remains high [18]. Clinically, approximately 80% of AP patients have mild disease and do not need any specific treatment, while approximately 20% of AP patients develop severe disease combined with local or systemic complications and even death [1]. At present, there is no specific treatment for AP; therefore, further research on AP pathogenesis is necessary. Inflammatory cytokines play an important role in the progression of mild acute pancreatitis (MAP) to SAP, according to numerous studies on the pathogenesis and treatment of SAP [19, 20]. Therefore, to prevent the onset and progression of SAP, blocking inflammatory mediators early in the AP process and preventing pancreatic acinar cell injury are crucial. Pancreatic acinar cells are believed to produce enzymes that cause pancreatic injury in AP. Currently, the understanding of the pathogenesis of pancreatitis primarily focuses on research on acinar cells [21]. Therefore, establishing an AP model in acinar cells and conducting functional studies will help us understand the pathogenesis and molecular mechanism of AP. Pancreatic acinar cells are the main cell type of the pancreas and the primary target of pancreatic injury [22]. In our study, we used the pancreatic acinar cell line (AR42J) and LPS to establish an AP model in vitro. We constructed an animal model of TLCS-mediated pancreatitis and used it to investigate biochemical and histological changes. In AP, acinar cell injury occurs; resulting in the release of serum amylase from damaged acinar cells. Serum amylase levels increase rapidly 3–6 h after the onset of symptoms and continue for 3–5 days [23]. In the present study, the serum amylase concentration was significantly elevated in the AP group compared to the control group. Serum amylase levels were significantly lower in the VB6 group than in the AP group, and these findings were consistent with the findings in the cellular model.

VB6 is a water-soluble vitamin that was originally discovered in the 1930s, and PLP is its biologically active form [24]. It is involved in a wide range of metabolic, physiological and developmental processes. VB6 can be found in a variety of foods, and people are unable to synthesize this important micronutrient. It exists in both nonphosphorylated forms, which are absorbed through passive diffusion in the jejunum and ileum and phosphorylated variants that require a dephosphorylation reaction catalyzed by intestinal alkaline phosphatases for absorption [25]. In humans, PLP is primarily catabolized through the production of 4-pyridine polyoxic acid, which is mediated by aldehyde oxidase 1 and is subsequently excreted in the urine [26]. Many studies have shown that low plasma VB6 levels are associated with typical inflammatory chronic diseases such as rheumatoid arthritis (RA) [27] and IBD [28]. It is also related to an increased risk of coronary artery disease (CVD) [29] and is negatively correlated with inflammatory marker levels. Moreover, VB6 inhibits acute pneumonia in mice by inhibiting macrophage activation [30]. However, little is known about the relationship between VB6 and AP. Our study is the first to report the potential beneficial effects of VB6 in combination with AP. In our study, TLCS significantly increased the serum amylase and lipase levels, which are good indicators for the diagnosis of AP [31]. These findings clearly indicate that VB6 attenuates pathological damage to the pancreas; reduces the serum amylase and lipase levels; and decreases the levels of the proinflammatory factors TNF-α, IL-1β and IL-6 in the rat AP model. This suggests that VB6 reduces pancreatic injury. Similarly, in the LPS-stimulated AR42J cell line, VB6 reduced acinar cell damage; and increased cell viability, as determined by the CCK8 assay. These results confirmed that VB6 protects PACs in multiple aspects and reduces the inflammatory response in AP.

Myeloperoxidase (MPO) is a member of the haem peroxidase superfamily. It is often used as a biomarker for activated neutrophils and is characterized by powerful oxidative and proinflammatory properties. During AP, neutrophils act as the first line of defense against tissue damage, and cytokines are expressed and released as part of the immune response. An increase in MPO activity indicates the activation of neutrophils and the severity of the damage caused by AP [32]. In the present study, treatment with TLCS resulted in a significant increase in the number of MPO-positive cells, indicating neutrophil infiltration in the pancreas. However, treatment with VB6 significantly reduced pancreatic neutrophil infiltration. In summary, we believe that VB6 can reduce the release of inflammatory cytokines and the recruitment of neutrophils, thus attenuating the tissue damage caused by TLCS. The results of in vitro experiments further showed that VB6 significantly inhibited the expression of MPO in pancreatic acinar cells.

There is a well-established connection between AP and Endoplasmic reticulum (ER) stress, with ER stress playing a crucial role in driving the progression of AP [33]. When misfolded proteins accumulate in the ER due to external stimuli, it can cause excessive aggregation, leading to ER stress and potentially triggering apoptosis if the stress continues. However, when ER stress is severe or prolonged, the UPR switches from a pro-survival to a pro-apoptotic signal, committing the cell to death [34]. Bax and Bcl-2 are regulated in mitochondria-dependent apoptotic pathways, activating caspase-3 and contributing to apoptosis [35]. In the present study, after both in vivo and in vitro induction of AP, we found an increase in the expression of Bax and decreased expression of Bcl-2, an antiapoptotic protein negatively regulated by bax [31]. Moreover, this trend was reversed after the use of VB6. Our study is consistent with previous research that suggested the inhibition of pancreatic apoptosis by Nimbolide may be responsible for the amelioration of AP in vivo and in vitro [36]. Caspase-3, a member of the caspase family, plays a crucial role in the execution of apoptosis [37]. It is activated in apoptotic cells through both extrinsic (death ligands) and intrinsic (mitochondrial) pathways [38]. The mitochondrial pro-apoptotic pathway leads to the permeability of the outer mitochondrial membrane and the release of apoptotic factors, including cytochrome c, from the mitochondrial intermembrane space into the cytoplasm. Subsequently, an apoptotic vesicle complex is formed, which then triggers the activation of effector cysteine asparaginases, including caspase-3 and caspase-7 [39]. During the process of apoptosis, the activation of caspase-3 results in the cleavage of various downstream substrates, ultimately leading to characteristic morphological alterations in apoptotic cells [40]. In their research, Zhao et al. [41] demonstrated the involvement of the Akt/caspase-3 signaling pathway in the regulation of cell apoptosis in hepatocellular carcinoma. In our own investigation, we observed an increase in caspase-3 levels in the AP group, whereas the expression of caspase-3 was reduced upon administration of VB6. Therefore, we propose that VB6 may influence the apoptosis of pancreatic acinar cells through the caspase-3 pathway. VB6 is a commonly used rehydration element in the clinic. In the future, we can observe the improvement in the disease course and changes in inflammatory factor levels in AP patients treated with or without VB6 and perform a preliminary evaluation of clinical treatment.

In summary, this study conducted a preliminary investigation into the potential of VB6 to modulate apoptosis and impede the progression of AP via the caspase3 signaling pathway. It is postulated that the inhibition of inflammation and the decrease in inflammatory cytokines play a role in diminishing the proportion of apoptotic acinar cells. The findings of this study offer a novel standpoint for the management of AP. Although our study confirmed the protective effect of VB6, there are still several limitations, and research on the underlying mechanism is relatively limited. However, further studies are needed to explore the mechanism of action of VB6.

## Conclusion

The findings of this study demonstrated that treatment with VB6 effectively alleviated the severity of AP both in vivo and in vitro, as evidenced by reduced pancreatic damage and improved cell viability. The underlying mechanism of this therapeutic effect may be attributed to the inhibition of the caspase3 signaling pathway by VB6. Furthermore, the beneficial effect of VB6 treatment could be attributed to its ability to suppress the inflammatory response and cell apoptosis. These results have significant implications for the identification of potential therapeutic targets for the treatment and prevention of AP, providing a valuable theoretical foundation for further research in this area.

### Electronic supplementary material

Below is the link to the electronic supplementary material.

Supplementary Material 1

## Data Availability

The datasets used and/or analysed during the current study available from the corresponding author on reasonable request.
