# Supplementary material for: Vitamin B6 ameliorates acute pancreatitis by suppressing the caspase3 signaling pathway
Source: BMC Gastroenterol. 2024 May 2;24:151. doi: 10.1186/s12876-024-03248-1 (PMC11067178; doi:10.1186/s12876-024-03248-1)
Supplement: Supplementary file 1 — Supplementary Material 1 [file 12876_2024_3248_MOESM1_ESM.pdf]

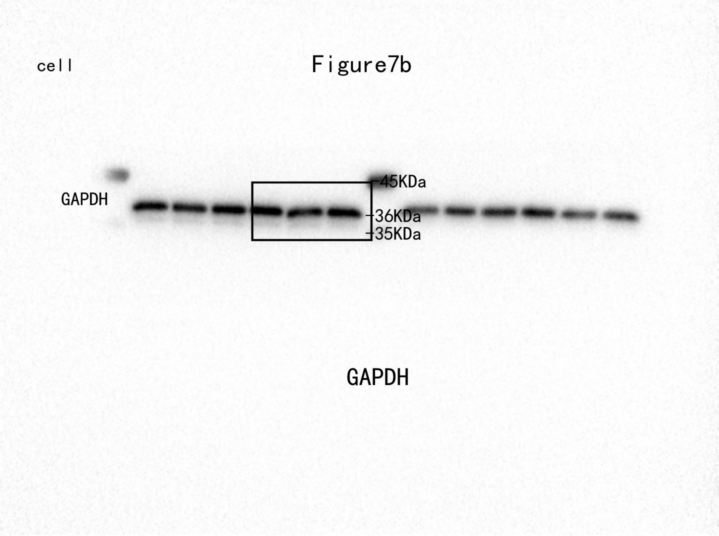
cell
Figure7b
GAPDH
45KDa
36KDa
35KDa
GAPDH

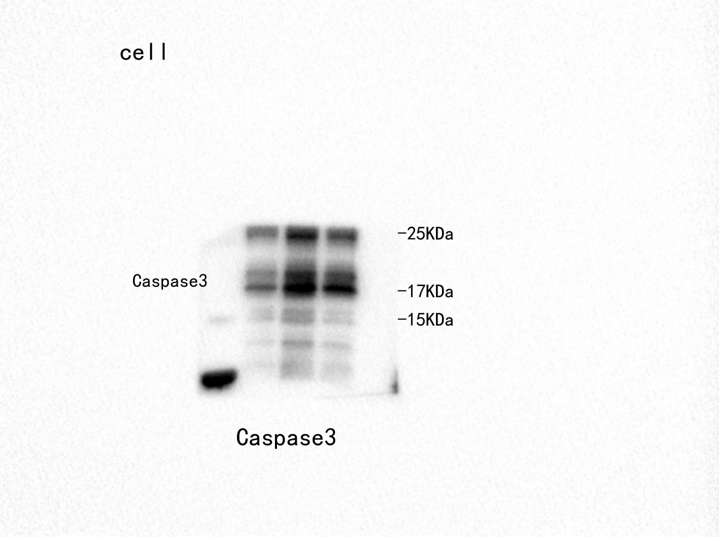
cell
Caspase3
-25KDa
-17KDa
-15KDa
Caspase3

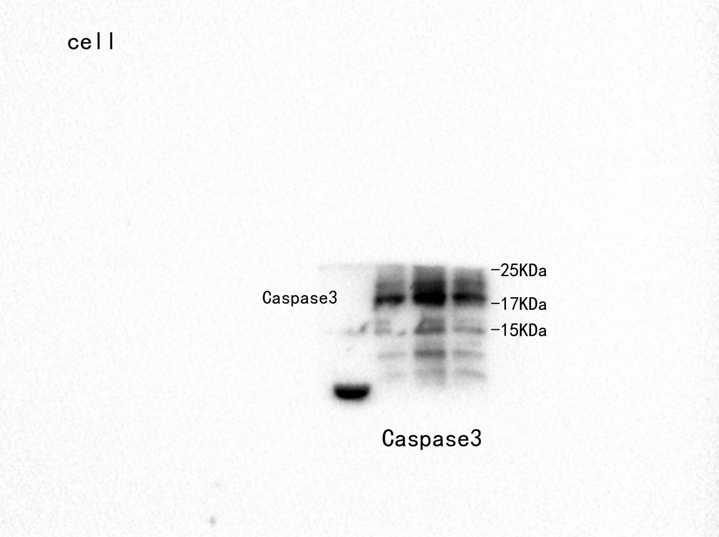
cell
Caspase3
-25KDa
-17KDa
-15KDa
Caspase3

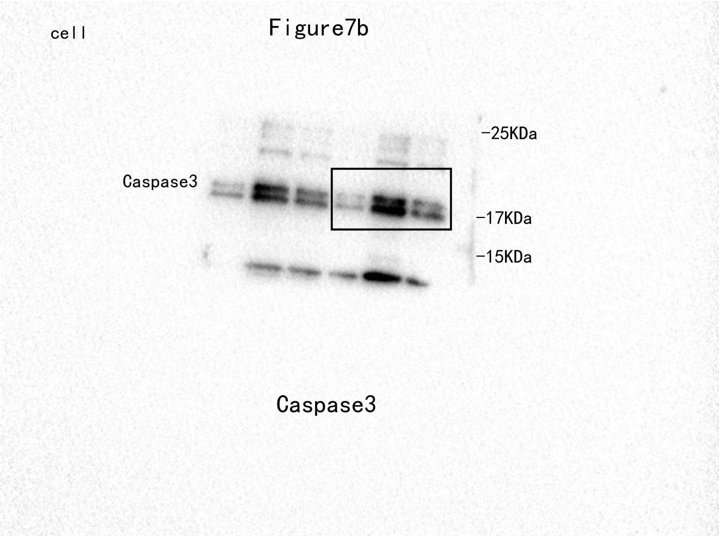
cell
Figure7b
Caspase3
-25KDa
-17KDa
-15KDa
Caspase3

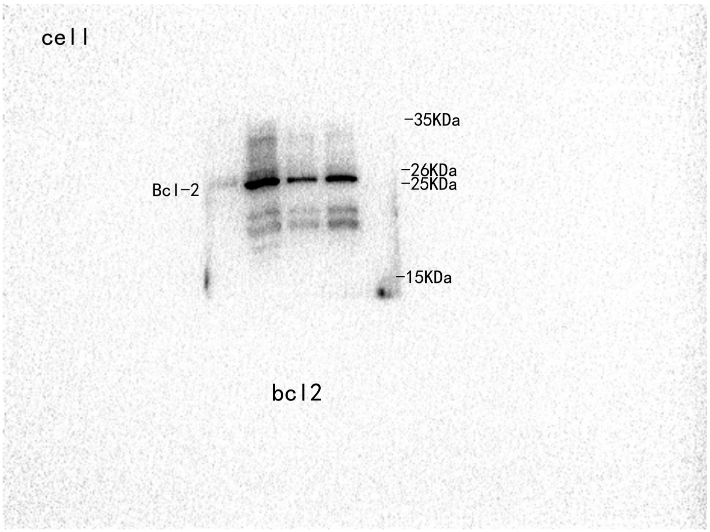
cell
-35KDa
Bcl-2
-26KDa
-25KDa
-15KDa
bcl2

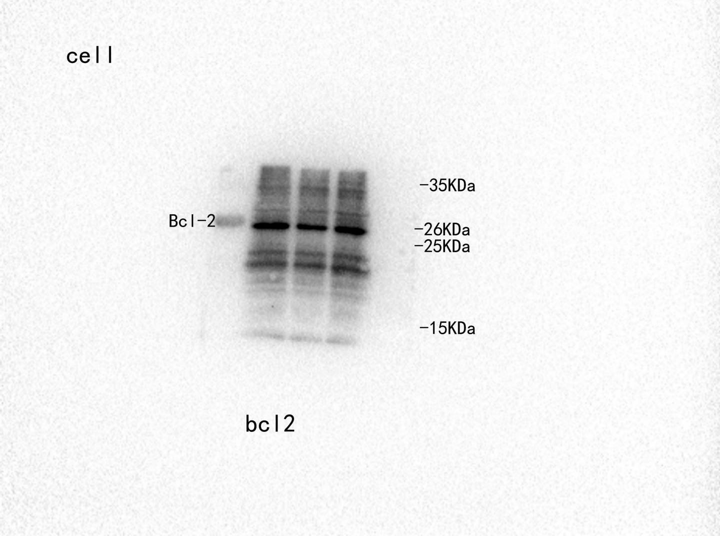
cell
-35KDa
Bcl-2
-26KDa
-25KDa
-15KDa
bcl2

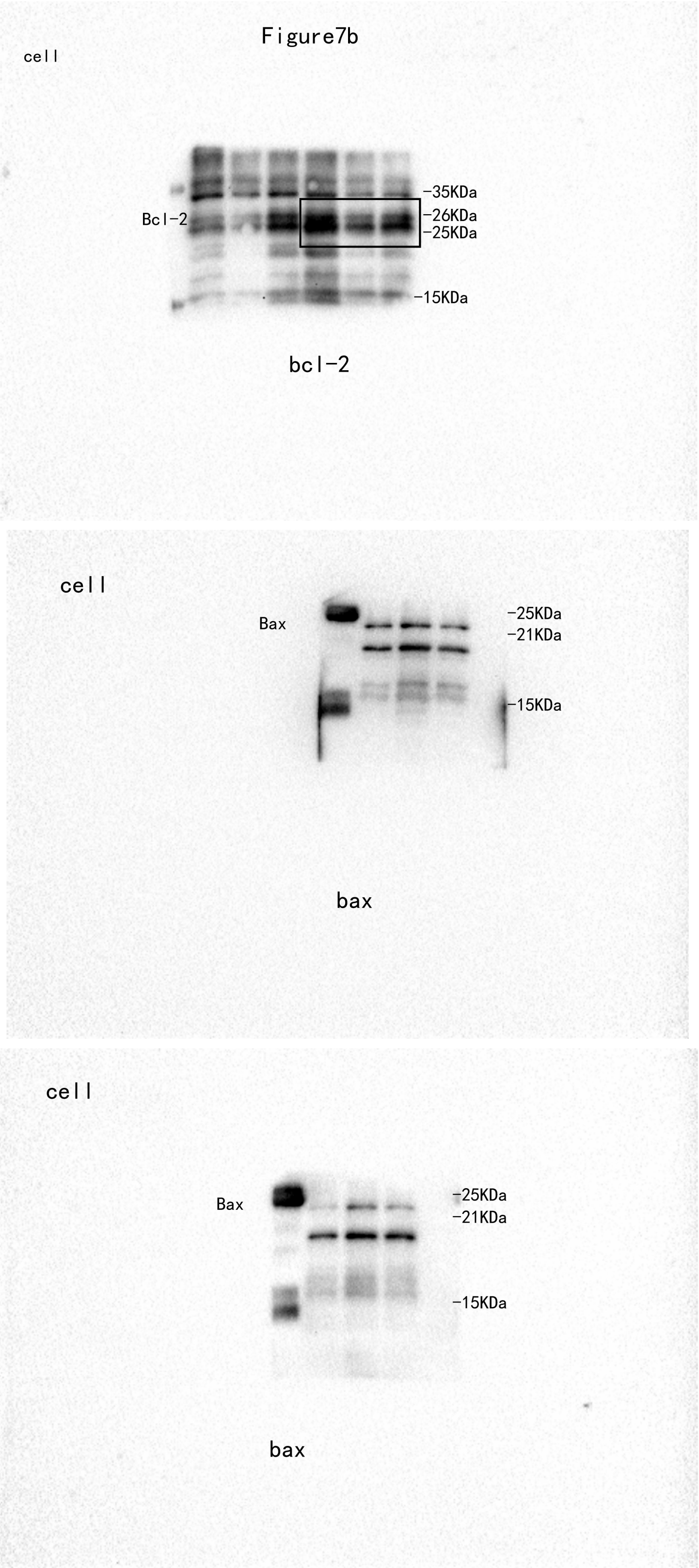
Figure7b
cell
Bcl-2
-35KDa
-26KDa
-25KDa
-15KDa
bcl-2
cell
Bax
-25KDa
-21KDa
-15KDa
bax
cell
Bax
-25KDa
-21KDa
-15KDa
bax

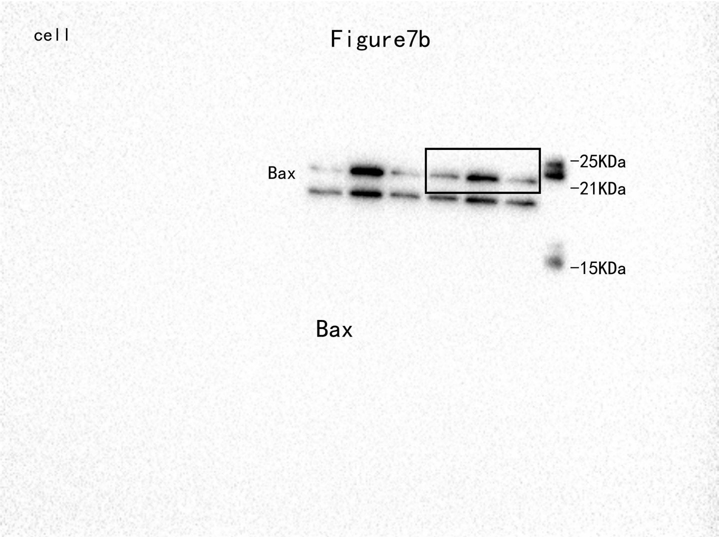
cell
Figure7b
Bax
-25KDa
-21KDa
-15KDa
Bax

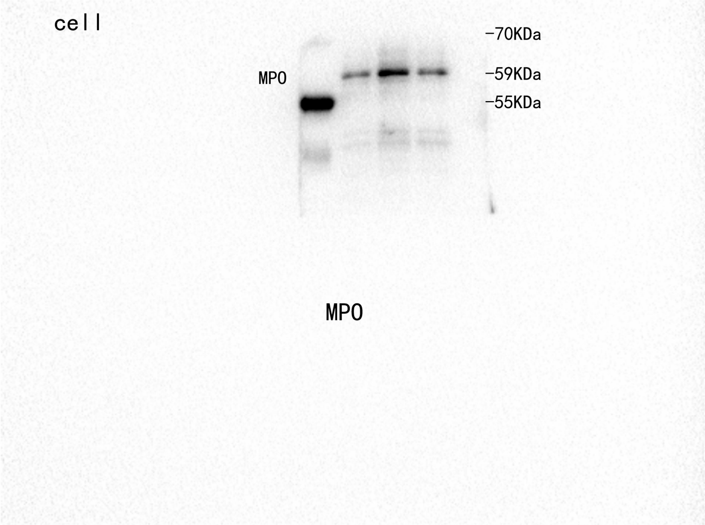
cell
MPO
-70KDa
-59KDa
-55KDa
MPO

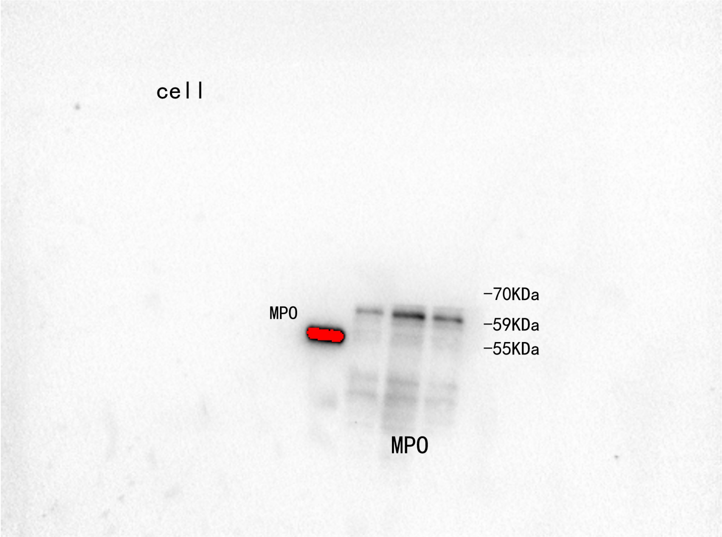
cell
MPO
-70KDa
-59KDa
-55KDa
MPO

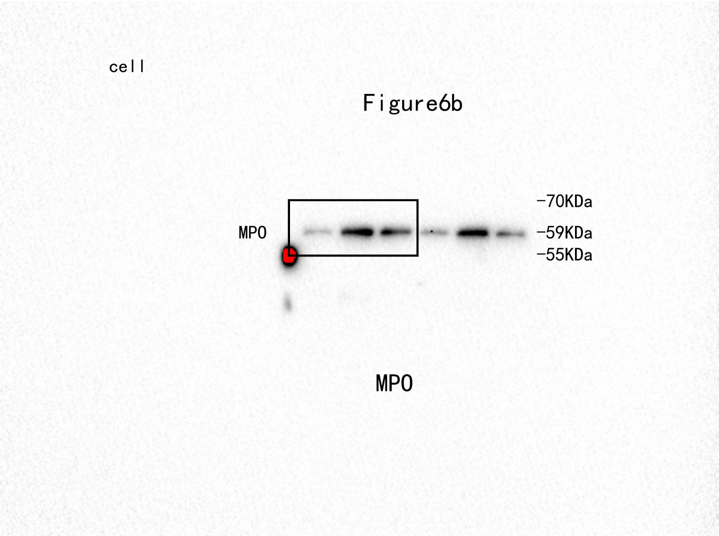
cell
Figure6b
MPO
-70KDa
-59KDa
-55KDa
MPO

cell

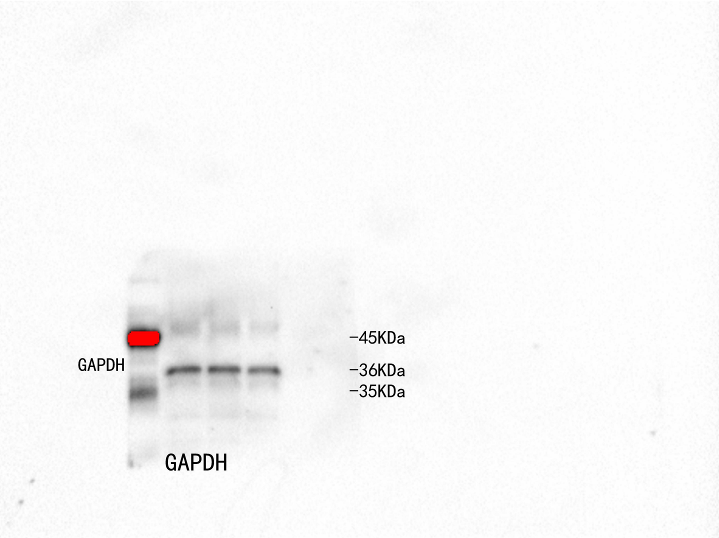
GAPDH
-45KDa
-36KDa
-35KDa
GAPDH

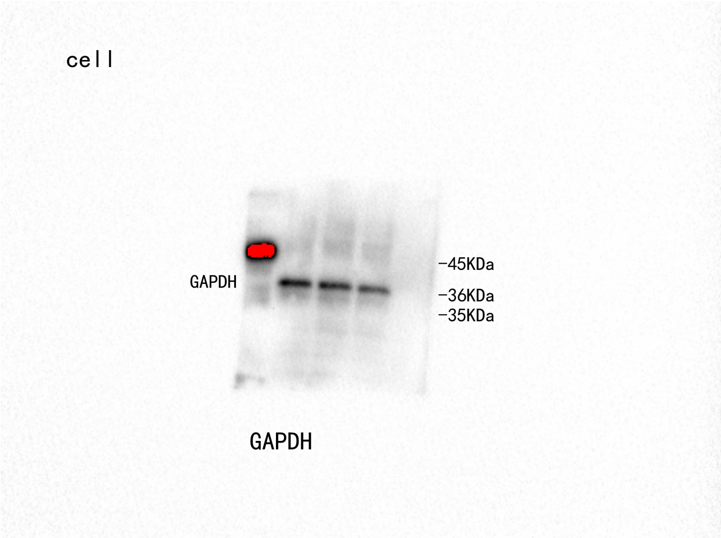
cell
GAPDH
-45KDa
-36KDa
-35KDa
GAPDH

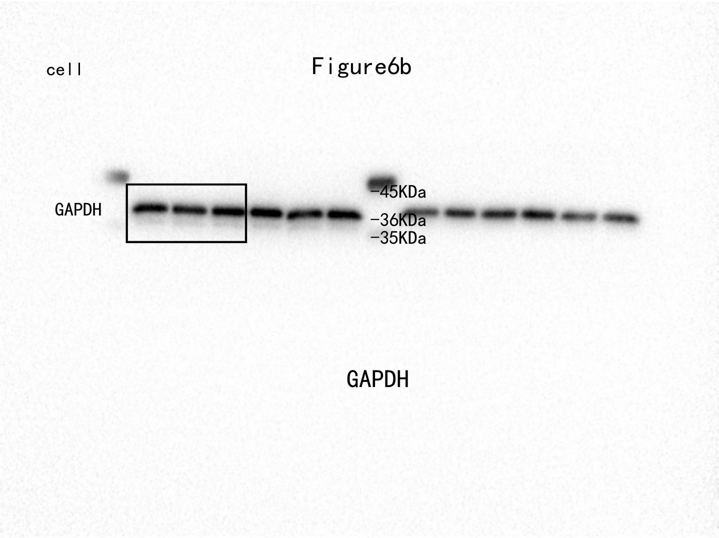
cell
Figure6b
GAPDH
-45KDa
-36KDa
-35KDa
GAPDH

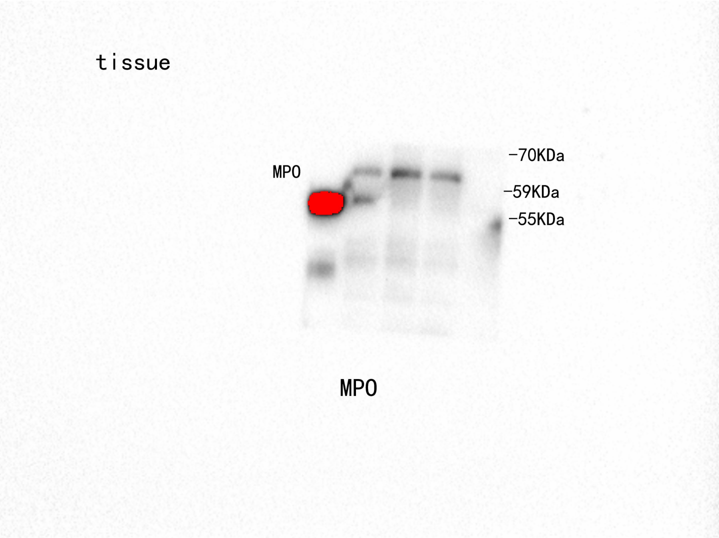
tissue
MPO
-70KDa
-59KDa
-55KDa
MPO

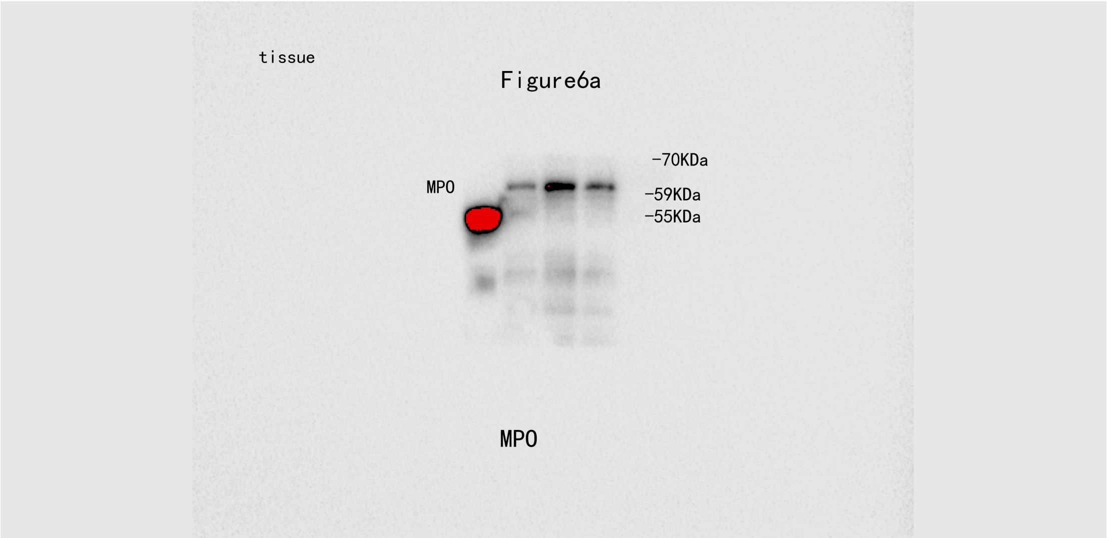
tissue
Figure6a
MPO
-70KDa
-59KDa
-55KDa
MPO

tissue

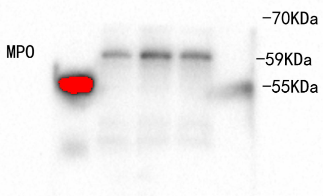

MPO

tissue

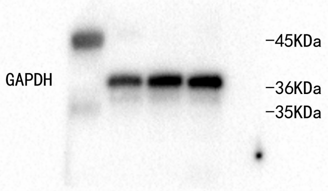

GAPDH

Figure6a

tissue

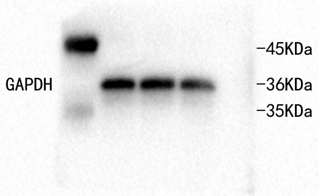

GAPDH

tissue

Figure7a

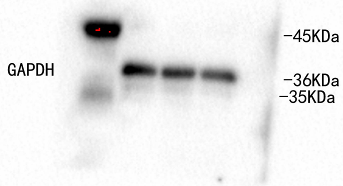

GAPDH

tissue

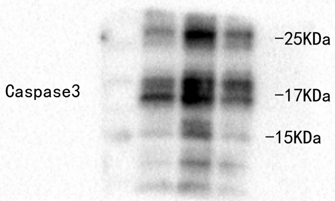

caspase3

tissue

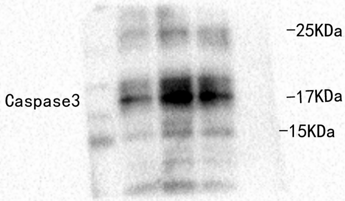

Caspase3

Figure7a

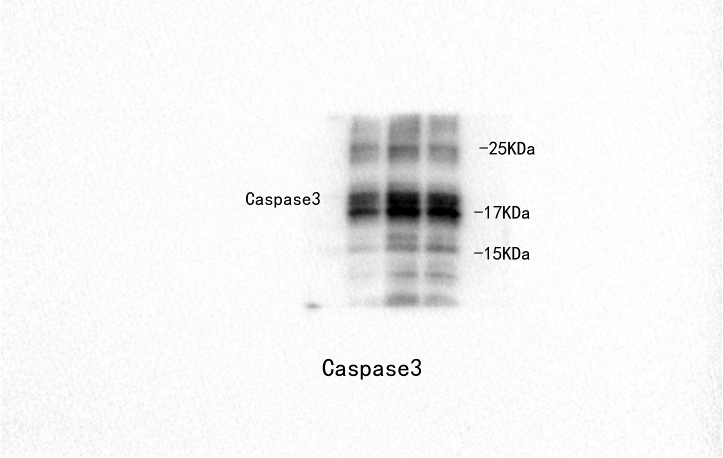
tissue
Caspase3
-25KDa
-17KDa
-15KDa
Caspase3

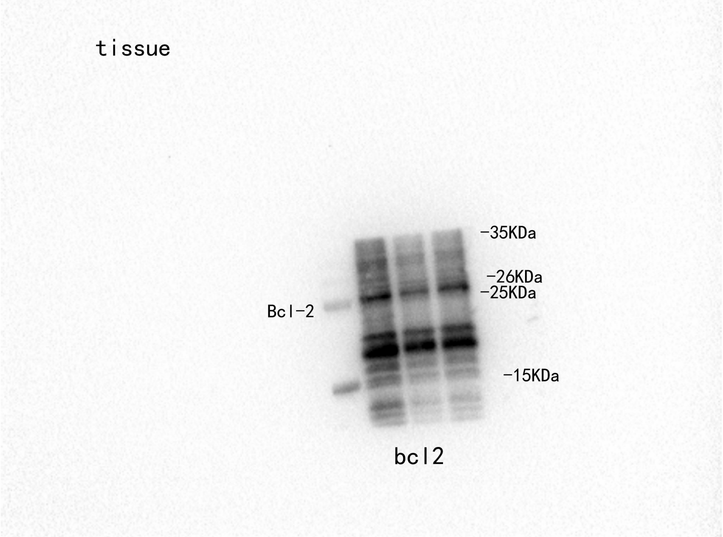
tissue
-35KDa
-26KDa
-25KDa
Bcl-2
-15KDa
bcl2

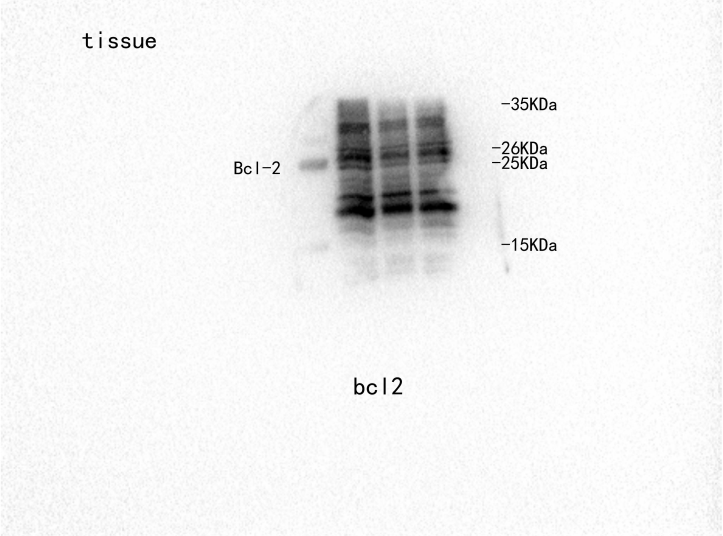
tissue
-35KDa
-26KDa
-25KDa
Bcl-2
-15KDa
bcl2

tissue

Figure7a

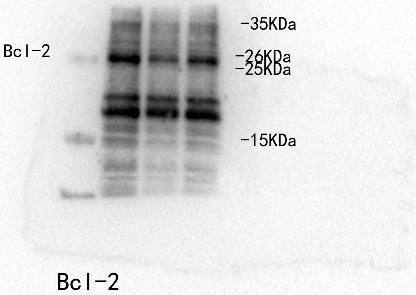

Bcl-2

tissue

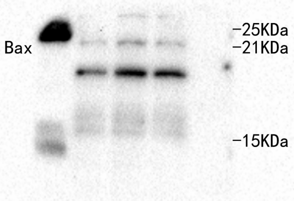

bax

tissue

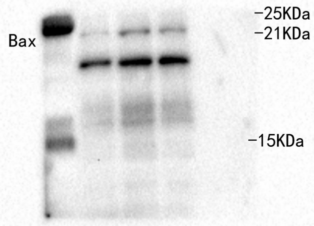

bax

Figure7a

tissue

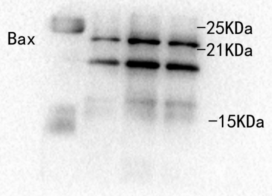

Bax
